# Supplementary material for: Bayesian refinement of protein structures and ensembles against SAXS data using molecular dynamics
Source: PLoS Comput Biol. 2017 Oct 18;13(10):e1005800. doi: 10.1371/journal.pcbi.1005800 (PMC5662244; doi:10.1371/journal.pcbi.1005800)
Supplement: S1 Archive — (BZ2) [file pcbi.1005800.s011.tar.bz2 › gromacs_4.62_bayesian_saxs_refi/share/html/online/xvg.html]

xvgr


## GRACE/XMGR, The ultimate plotting program

|  |  |
| --- | --- |
| Main Table of Contents | **VERSION 4.6** |
| GROMACS homepage | **Sat 19 Jan 2013** |

---

### Description

Almost all output from GROMACS analysis tools is ready as input for
Grace, formerly known as Xmgr. We use Grace, because it is very flexible, and it is also
free software. It produces PostScript(tm) output, which is very suitable
for inclusion in eg. LaTeX documents, but also for other word processors.

A sample Grace session with GROMACS data is shown below.  
  
   

---

http://www.gromacs.org
